# RETRACTED: Osman et al. Lipolytic Postbiotic from *Lactobacillus paracasei* Manages Metabolic Syndrome in Albino Wistar Rats. *Molecules* 2021, *26*, 472

**DOI:** 10.3390/molecules31081286

**Published:** 2026-04-15

**Authors:** Ali Osman, Nashwa El-Gazzar, Taghreed N. Almanaa, Abdalla El-Hadary, Mahmoud Sitohy

**Affiliations:** 1 Biochemistry Department, Faculty of Agriculture, Zagazig University, Zagazig 44511, Egypt; 2 Botany and Microbiology Department, Faculty of Science, Zagazig University, Zagazig 44519, Egypt; 3 Department of Botany and Microbiology, College of Science, King Saud University, Riyadh 11451, Saudi Arabia; 4 Biochemistry Department, Faculty of Agriculture, Benha University, Benha 13736, Egypt

The Journal retracts the article “Lipolytic Postbiotic from *Lactobacillus paracasei* Manages Metabolic Syndrome in Albino Wistar Rats” [1] cited above.

Following publication, concerns were brought to the attention of the Editorial Office regarding the integrity of images presented in this publication [1].

In accordance with standard journal procedures, the Editorial Office and Editorial Board conducted an investigation that identified indications of inappropriate image editing and duplication of panels between figures in this article [1] and a previously published article [2] by a different group of authors. These concerns relate to Figure 5B, where the PC and ATOR lanes appear to be identical, and to Figure 7, where plate 5 sub-images are duplicated in Figure 6A,B of article [2], despite being described under different experimental conditions. Although the authors cooperated during the investigation, the explanations and supporting materials provided were not deemed sufficient by the Editorial Board to resolve these concerns. Consequently, the Editorial Board has lost confidence in the reliability of the findings and decided to retract this publication [1], as per MDPI’s retraction policy.

This retraction was approved by the Editor-in-Chief of the journal *Molecules*.

The authors have been informed of this retraction and have indicated their disagreement with this decision.
